# The counseling african americans to control hypertension (caatch) trial: baseline demographic, clinical, psychosocial, and behavioral characteristics

**DOI:** 10.1186/1748-5908-6-100

**Published:** 2011-09-01

**Authors:** Senaida Fernandez, Jonathan N Tobin, Andrea Cassells, Marleny Diaz-Gloster, Chamanara Kalida, Gbenga Ogedegbe

**Affiliations:** 1 Center for Healthful Behavior Change, Division of General Internal Medicine, Department of Medicine, New York University School of Medicine, 550 First Avenue, New York, NY, USA; 2 Clinical Directors Network (CDN), 5 West 37th Street, New York, NY, USA; 3 Department of Epidemiology and Population Health, Albert Einstein College of Medicine of Yeshiva University, 1300 Morris Park Avenue, Bronx, NY, USA

## Abstract

**Background:**

Effectiveness of combined physician and patient-level interventions for blood pressure (BP) control in low-income, hypertensive African Americans with multiple co-morbid conditions remains largely untested in community-based primary care practices. Demographic, clinical, psychosocial, and behavioral characteristics of participants in the Counseling African American to Control Hypertension (CAATCH) Trial are described. CAATCH evaluates the effectiveness of a multi-level, multi-component, evidence-based intervention compared with usual care (UC) in improving BP control among poorly controlled hypertensive African Americans who receive primary care in Community Health Centers (CHCs).

**Methods:**

Participants included 1,039 hypertensive African Americans receiving care in 30 CHCs in the New York Metropolitan area. Baseline data on participant demographic, clinical (*e.g*., BP, anti-hypertensive medications), psychosocial (*e.g*., depression, medication adherence, self-efficacy), and behavioral (*e.g*., exercise, diet) characteristics were gathered through direct observation, chart review, and interview.

**Results:**

The sample was primarily female (71.6%), middle-aged (mean age = 56.9 ± 12.1 years), high school educated (62.4%), low-income (72.4% reporting less than $20,000/year income), and received Medicaid (35.9%) or Medicare (12.6%). Mean systolic and diastolic BP were 150.7 ± 16.7 mm Hg and 91.0 ± 10.6 mm Hg, respectively. Participants were prescribed an average of 2.5 ± 1.9 antihypertensive medications; 54.8% were on a diuretic; 33.8% were on a beta blocker; 41.9% were on calcium channel blockers; 64.8% were on angiotensin converting enzyme (ACE) inhibitors/angiotensin receptor blockers (ARBs). One-quarter (25.6%) of the sample had resistant hypertension; one-half (55.7%) reported medication non-adherence. Most (79.7%) reported one or more co-morbid medical conditions. The majority of the patients had a Charlson Co-morbidity score ≥ 2. Diabetes mellitus was common (35.8%), and moderate/severe depression was present in 16% of participants. Participants were sedentary (835.3  ± 1,644.2 Kcal burned per week), obese (59.7%), and had poor global physical health, poor eating habits, high health literacy, and good overall mental health.

**Conclusions:**

A majority of patients in the CAATCH trial exhibited adverse lifestyle behaviors, and had significant medical and psychosocial barriers to adequate BP control. Trial outcomes will shed light on the effectiveness of evidence-based interventions for BP control when implemented in real-world medical settings that serve high numbers of low-income hypertensive African-Americans with multiple co-morbidity and significant barriers to behavior change.

## Background

Hypertension affects approximately 65 million adults in the United States [1], and disproportionately affects African Americans in terms of prevalence, treatment, and control rates compared to whites [2,3]. These disparities may explain the poorer adverse hypertension-related outcomes in African Americans [4]. The cardiovascular benefits of blood pressure (BP) control are well-documented [5], as are the utility of patient- and provider-centered interventions in promoting BP control [6-9]. Lifestyle interventions of reduced sodium intake [7,10] and other dietary modifications (increased consumption of fiber, fruits, vegetables, and low fat dairy; reduced consumption of saturated and total fat) [11], and increased physical activity and weight loss [6] have resulted in significant BP reduction. While these interventions have proven efficacious in clinical trials, and although there are many community-based interventions that engage African Americans (and are often conducted in low-income communities), their effectiveness remains largely untested among hypertensive African Americans who receive care in low-resource settings such as Community Health Centers (CHCs).

The Counseling African Americans to Control Hypertension (CAATCH) trial [12] was designed to evaluate, in a cluster randomized trial, the effectiveness of a multi-level, multi-component, evidence-based intervention compared to usual care (UC) in improving BP control in hypertensive African Americans who receive care in CHCs. The trial targets barriers to optimal hypertension control at the patient, provider, and practice levels in a large cohort of African American adults (n = 1,039) [12]. As such, this trial has the potential to expand our understanding of barriers and facilitators of intervention implementation in 'real-world' clinical settings, as well as the effectiveness of lifestyle interventions in previously understudied and underserved populations. This paper describes the demographic, clinical, psychosocial, and behavioral characteristics of CAATCH trial participants in order to highlight some of the patient-related barriers to hypertension control present in this sample.

## Methods

### Study Design and Setting

A detailed description of the rationale, design, and methods of this trial has been published elsewhere [12]. The study protocol was approved by the Institutional Review Boards (IRBs) of Columbia University, New York University, and Clinical Directors Network (CDN), and all participants provided informed consent to participate. Briefly, CAATCH is a two-arm, cluster-randomized controlled trial (RCT) implemented in 30 CHCs, with 15 sites randomly assigned to the intervention condition (IC) and 15 to UC.

### Participants

**Figure 1:**
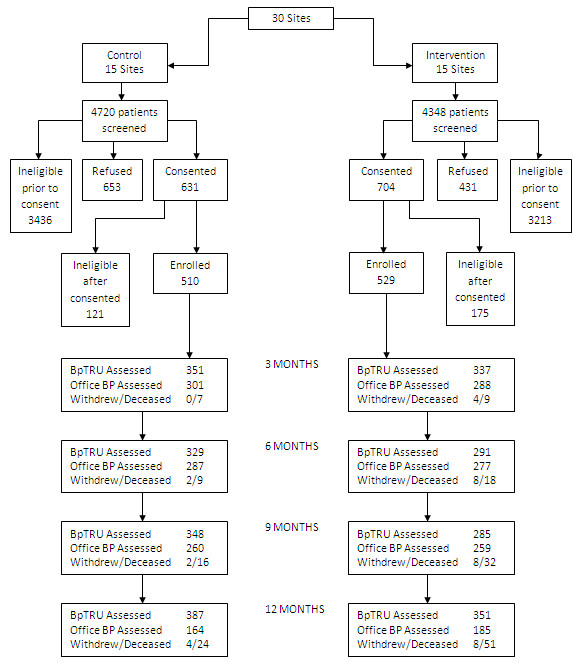
**Flow of participants through study**.

The participants included 1,039 African American adult men and women. Patients were eligible for the study if they: self-identified as black or African American; were at least 18 years old, were receiving care at the participating CHC for at least six months prior to enrollment; had a diagnosis of hypertension (HTN), were taking at least one anti-hypertensive medication, and had uncontrolled BP at the time of enrollment based on standardized measurement at study visit (systolic BP (SBP) ≥ 140 mm Hg or diastolic BP (DBP) ≥ 90 mmHg; for those with diabetes or kidney disease, SBP ≥ 130 mm Hg or DBP ≥ 80 mm Hg). Additional details on inclusion and exclusion criteria have been published elsewhere [12]. Figure 1 shows the flow of patients through the study in a CONSORT [13] diagram.

### Interventions

The intervention was implemented at the practice level, such that all physicians and patients at a given site were in the same treatment condition: IC or UC. Within the IC sites, the CAATCH intervention is comprised of three components targeted at patients (interactive computerized hypertension education, home BP monitoring, and monthly behavioral counseling on lifestyle modification) and two components targeted at physicians (monthly CME-accredited case rounds based on JNC-7 guidelines [5], chart audit and provision of feedback on clinical performance, and patients' home BP readings). Additional details on the intervention and its delivery are described elsewhere [12].

### Objectives

The goal of CAATCH was to evaluate, in cluster randomized trial, the effectiveness of a multi-level, multi-component, evidence-based intervention compared to UC in improving BP control among hypertensive African Americans who receive care in CHCs. We hypothesize that patients randomized to the IC will have, compared to those in the UC condition: a higher BP control rate at 12 months; greater reduction in both SBP and DBP at 12 months; and a higher rate of maintenance of intervention effect one year after completion of the trial. In addition, the IC will be more cost-effective in improving BP control rate at 12 months compared to UC.

### Outcomes

The primary outcome was the proportion of patients with adequate BP control at 12 months; and the maintenance of intervention effects one year after the trial. BP was defined as uncontrolled if the average BP ≥ 140 mm Hg or DBP ≥ 90 mm Hg (for those without comorbidity) OR average SBP ≥ 130 mm Hg or DBP ≥ 80 mm Hg (for those with diabetes or kidney disease). The secondary outcomes are within-patient change in BP from baseline to 12 months, and the cost effectiveness of intervention at 12 months.

### Data collection

Data collection and study measurements were performed by trained research assistants (RAs) via interviews, direct measurements, and chart review. A complete listing of all measures and details of assessment protocol has been published elsewhere [12]. At baseline, three BP readings were taken by trained RAs using a validated automated BP monitor (BPTru, Model BPM-300; BpTRU Medical Devices, Coquitlam, BC, Canada) with the patient seated comfortably and following American Heart Association (AHA) guidelines [14]. The average of the three readings was used as the measure for baseline assessment. Psychosocial and behavioral characteristics were assessed with validated self-report measures and included medication adherence (4-item Morisky Scale) [15], self-efficacy (Medication Adherence Self-Efficacy Scale; MASES) [16], health literacy (Rapid Estimate of Adult Literacy in Medicine; REALM) [17], dietary intake (Rapid Eating Assessment for Patients; REAP) [18], physical activity (Paffenbarger Physical Activity Scale) [19], daytime sleepiness (Epworth Sleepiness Scale) [20], depression (Patient Health Questionnaire-9; PHQ-9) [21], and general physical and mental health (version one of the 12-Item Short Form Health Survey; SF-12) [22]. At the CHC site level, data were gathered on whether CHCs utilized electronic health records (EHRs), and whether they were part of the Health Resources and Services Administration (HRSA) Health Disparities Collaboratives http://www.healthcarecommunities.org. The HRSA sponsored Health Disparities Collaboratives (HDCs) use the structure of the Chronic Care Model (CCM), which is an organizational approach to caring for people with chronic disease who are seen in a primary care setting. The system is population-based, and creates practical, supportive, evidence-based interactions between an informed, activated patient and a prepared, proactive practice team; it identifies six major categories that must be addressed to achieve substantial change, including the healthcare organization, community resources and policies, self-management support, decision support, delivery system design, and clinical information systems [23].

### Power analysis and sample size

The design for the CAATCH trial was based on power calculations for the proposed analysis of differential changes in office SBP and DBP between IC and UC sites (CHCs). These calculations were based on post-intervention data available from Phase one of the medication Adherence and Blood Pressure Control (ABC) trial, which was conducted by this research group in several of the same CHCs [24]. BPs for those in the ABC trial home blood pressure monitoring (HBPM) intervention (n = 137) were compared to those in the UC group (n = 80) in nine sites, and office BPs decreased substantially in both conditions (SBP/DBP decreased 12.0/6.0 mmHg in HBPM versus 8.7/3.8 mmHg in UC). Of note, they decreased more (3.3/2.2 mmHg, *p *= 0.053/0.078) in the HBPM condition. Clustered sampling due to patients being nested within sites was controlled for, yielding estimates of the between-site and within-site variances in treatment effect (5.13 and 154.21 for SBP, and 1.72 and 86.35 for DBP). Sites were the primary unit of analysis, given that sites are randomized to treatment condition. To estimate the number of sites required, the within-treatment, between-site variance of site-level mean change scores was first estimated. This is estimated as the between-site (true) variance plus the sampling variability of the site mean. For SBP, this was 5.13 ± 154.21/Nsite, and for DBP it was 1.72 ± 86.35/Nsite, where Nsite is the number of patients per site. We initially planned for 30 patients per site, yielding variance estimates of 10.28 and 4.60 for site-level mean changes in SBP and DBP; taking square roots gives standard deviation estimates of 3.21 and 2.14. These estimates and the usual power calculations for a t-test were used to determine the number of sites needed to have adequate power to detect a specified effect size. With a total of 30 sites (with 30 patients at each site), 15 assigned to each condition (IC and UC), these estimates implied that there would be 80% and 81% power to detect SBP and DBP treatment effects equal to the HBPM effect reported above (using a 2-tailed, 0.05-level test). Because of the multifaceted nature of the intervention that was implemented in this study, it is reasonable to anticipate treatment effects of at least 4 mm Hg for SBP and 3 mm Hg for DBP. The estimated power to detect effects of this magnitude is 91% and 96%, respectively. Thus, based on this power analysis, we planned to randomize 30 sites (1:1). Because we anticipated approximately a 15% rate of attrition, the enrollment target was set at 1,058 patients from these sites, which would yield a final sample of 900 patients who complete the study (an average of 30 per site).

### Analytic plan

The CAATCH trial has three nested levels of sampling: site, physician, and patient. Sites were matched for size (large/medium versus small), creating 15 matched pairs. Within each pair, one site was randomly assigned to IC and the other to UC. Due to a variety of unspecified factors, it was assumed that patients from the same site, and perhaps also patients having the same physician, would be somewhat more similar than randomly selected patients attending different CHCs or having different physicians. This implies correlated residuals due to 'clustered sampling,' that will be controlled in the primary outcome analysis by treating both CHC and clinician within CHC as random factors. Maximum likelihood estimates, approximate standard errors, and multi-level modeling statistical tests for primary outcome analysis will be obtained using PROC MIXED (SAS) [25,26]. The comparison of treatment groups with respect to dichotomous or ordinal measures (*e.g*., gender or smoker) will be performed using the MIXOR software [27,28], which estimates a logistic regression (with random effects). The clustered sampling effects associated with CHC and physician will be adjusted for in the equation. As appropriate, the model will be further augmented to include both person-level covariates (*e.g*., gender) and time-varying covariates (*e.g*., body mass index). As stated, the primary hypothesis concerns the treatment by time interaction.

## Results

### CHC characteristics

The 30 CHCs serve primarily low-income, African American populations. The median household yearly income in CHC zip codes was $32,499 (SD = $17,054) and mean percentage of African American adults in CHC zip codes was 53% (SD = 27%). A majority of the sites in the trial were located in metropolitan New York City (NYC) (73.30%), upstate New York (13.30%), and northern New Jersey (13.30%). The IC and UC sites were similar in characteristics (all *p*-values ≥ 0.05). The mean number of patients seen at the CHCs was 10,432 patients (SD = 13, 157) with 33.33% of sites classified as 'large' or 'extra large' CHC. Only a quarter of sites utilized EHRs, and less than one-half were participating in the HRSA Health Disparities Collaborative (see Table 1).

**Table 1:** Community Health Center (CHC) Characteristics at Baseline

Characteristic	Total (n = 30)	Intervention Group (n = 15)	Usual Care Group (n = 15)	*p*
NYC	73.3	66.7	80.00	0.08
Upstate NY	13.3	6.7	20.00	
NJ	13.3	26.7	0.00	
Size/#Users (SD)	10,432 (13,157)	9,311 (8,234)	11,553 (16,973)	0.65
% Large CHC	33.3	33.3	33.3	1.0
% Using Electronic Health Records (EHR)	26.7	20.0	33.3	0.41
% Participating in HRSA Health Disparities Collaboratives (HDC)	43.3	46.7	40.0	0.71
Median Household Income in Zip Code	32,499 (17,054)	28,352 (11,196)	36,645 (20,982)	0.19
Mean% African American Adults in Zip (SD)	52.6 (27.2)	57.1 (27.5)	48.3 (27.2)	0.38

*Note*. # Users = total number of patients across all divisions of care. HRSA = Health Resources and Services Administration.

### Clinician characteristics

CAATCH clinicians (n = 94) were middle-aged (*M *= 47.0 ± 14.1 years); 57% female; 43% African American, 24% White, 9% Hispanic; 57% were U.S. Born; had MD/DO degree (75%); had worked in their CHC an average of 8.0 ± 6.5 years. There were an average of 4.4 clinicians per CHC.

### Demographic characteristics

CAATCH participants were middle-aged (*M *= 56.9 ± 12.1 years), primarily female (71.6%), high-school educated (62.4%) and U.S. born (74.6%). Participants who were foreign-born had lived in the U.S. an average of 20 ± 12.4 years. One-half (54.0%) were insured through Medicaid and Medicare. As compared to UC, IC participants were more likely to be low-income (IC = 78.6% versus UC = 65.8%, *p <*0.01) and have no health insurance (IC = 11.3% versus UC = 3.4%, *p <*0.01; see Table 2).

**Table 2:** Demographic Characteristics of Participants at Baseline

Characteristic	Total (n = 1,039)	Intervention Group (n = 529)	Usual Care Group (n = 510)	*p*
Age in Years (mean ± sd; n = 1,026)	56.9 ± 12.1	56.7 ± 11.5	57.0 ± 12.8	0.72
Age ≥ 65 Years (%; n = 1,026)	26.5	26.8	26.2	0.85
Gender (%; n = 1,014)				
Female	71.6	69.3	74.0	0.10
Ethnicity (%; n = 953)				
Non-Hispanic	93.3	94.3	92.2	0.21
Hispanic	6.7	5.7	7.8	
Place of Birth (%; n = 941)				
USA	74.60	76.4	72.6	0.18
Non-USA	25.40	23.6	27.4	
Marital Status (%; n = 953)				
Separated/Divorced/Widowed	44.7	48.0	41.3	0.07
Married	25.8	25.5	26.1	
Never married	29.5	26.5	32.6	
Education Level (%; n = 956)				
≥ High School	62.4	59.9	65.2	0.14
< High School	36.9	39.7	34.0	
None	0.6	0.4	0.9	
Employment Status (%; n = 944)				
Not employed	11.0	12.3	9.6	0.15
Retired or Homemaker	55.2	57.1	53.2	
Employed: Part-time	13.6	12.1	15.1	
Employed: Full-time	20.2	18.5	22.1	
Income (%; n = 940)				
< $20,000/year	72.4	78.6	65.8	< 0.01**
≥ $20,000/year	27.6	21.4	34.2	
Insurance Status (%; n = 917)				
Medicaid	35.9	38.7	32.9	< 0.01**
Medicare	12.6	12.6	12.8	
Medicaid and Medicare	5.5	6.4	4.5	
Private/HMO	19.0	13.8	24.4	
None	7.4	11.3	3.4	
Other#	19.6	17.2	22.1	

*Note*. Data gathered through interview. #Category includes VA insurance; combination/multiple sources of coverage. *p < 0.05; **p < 0.01

### Clinical characteristics

Baseline mean SBP and DBP were 150.7 ± 16.7 mm Hg and 91.0 ± 10.6 mm Hg, respectively. SBP was significantly higher in the UC group (152.1 ± 16.8) compared to the IC group (149.3 ± 16.5, *p *= 0.007). There were no between group differences for DBP. Resistant hypertension, defined as any patient taking at least three antihypertensive medications including a diuretic, was present among 25.6% of participants. Over one-half of the participants were obese, and an additional one-quarter was overweight. Most (79.7%) reported one or more co-morbid conditions, with over one-half (55.1%) reporting two or more, and a sample mean Charlson Comorbidity score of 2.5 ± 2.5. Diabetes mellitus was the most commonly reported co-morbid condition (35.8%), and congestive heart failure (CHF) was more common among UC (13.5%) than IC (7.5%) participants (*p *= 0.002; See Table 3).

**Table 3:** Clinical Characteristics of Participants at Baseline

Characteristic	Total (n = 1,039)	Intervention Group (n = 529)	Usual Care Group (n = 510)	*p*
Baseline BP^ (mean ± sd; n = 1,039)				
SBP	150.7 ± 16.7	149.3 ± 16.5	152.1 ± 16.8	< 0.01**
DBP	91.0 ± 10.6	90.8 ± 10.7	91.2 ± 10.5	0.54
Resistant Hypertension† (%; n = 912)	25.6	27.8	23.6	0.15
Smoking Status† (%; n = 939)				
Current	29.8	32.0	27.5	0.08
Former	20.9	22.3	19.4	
Never	49.3	45.8	53.1	
Body Mass Index† (%; n = 647)				
Mean (SD), kg/m^2^	32.5 (7.9)	32.6 (7.6)	32.4 (8.4)	0.77
Overweight	26.3	28.3	23.2	0.15
Obese	59.7	59.9	59.3	
Number of Co-morbid Conditions‡ (%; n = 929)				
0	20.3	18.3	22.3	0.03*
1	24.6	28.1	20.9	
2 to 3	35.6	36.2	35.0	
> 3	19.5	17.3	21.8	
Comorbid Conditions‡ (%; n = 964)				
Charlson Score (mean ± sd; n = 965)	2.5 ± 2.5	2.4 ± 2.5	2.5 ± 2.5	0.70
Chronic Kidney Disease	2.5	2.4	2.6	0.87
Myocardial Infarction	7.4	6.9	7.9	0.53
Congestive Heart Failure	10.4	7.5	13.5	< 0.01**
Stroke	10.9	9.9	12.0	0.30
Diabetes Mellitus	35.8	37.2	34.3	0.35

*Note*. Data gathered through: ^direct observation, †chart review, ‡interview. BP = Blood Pressure; HTN = Hypertension. *p < 0.05; **p < 0.01

CAATCH participants were taking an average of 2.5 antihypertensive medications, with over one-third of the sample taking three or more medications. Over one-half of the participants were on a diuretic (alone or in combination with other drugs); the most common antihypertensive medication prescribed for patients in this study was ACE/ARBs with over two-thirds of the patients, and a little over one-half were prescribed diuretic alone on in combination with other drugs (see Table 4).

**Table 4:** Blood Pressure Medications at Baseline

	Total (n = 1,039)	Intervention Group (n = 529)	Usual Care Group (n = 510)	*p*
Number of Drugs in Hypertension Regimen (%; n = 907)				
1	32.7	30.1	35.8	
2	30.1	29.1	31.3	
3	18.0	20.4	15.1	
≥ 4	19.2	20.4	17.8	0.08
On a Diuretic (%; n = 912)	54.8	54.6	55.1	0.86
On a Beta Blocker (%; n = 912)	33.8	30.4	37.7	0.02*
If on a 2-Drug Regimen (%; n = 273), Percent on a Diuretic	57.9	57.3	58.5	0.85
If on a 3-Drug Regimen (%; n = 163), Percent on a Diuretic	63.2	65.0	60.3	0.55
If on a 3-Drug or Greater Regimen (n = 337), Percent on a Diuretic	70.0	68.5	72.3	0.46
If on a 2-drug Regimen (n = 273), Percent on ACE+CCB	6.2	6.3	6.2	0.96

*Note*. Data gathered through chart review.

### Psychosocial and behavioral characteristics

One-half of CAATCH participants reported medication non-adherence to their antihypertensive medication (55.7%), with a larger proportion of non-adherence in the UC group (63.1%) compared to the IC group (48.7%, *p <*0.01). Self-efficacy for medication taking was lower in the UC group (*M *= 2.2 ± 0.6) than the IC group (*M *= 2.3 ± 0.6, *p *= 0.02). With regards to health literacy, a smaller proportion of UC participants (54.1%), compared to IC participants (60.8%) demonstrated high health literacy (61 to 66 points range) on the REALM, (*p *= 0.04). Differences in eating behaviors were also found between groups, with IC participants engaging in slightly less healthy eating (*M *= 1.7 ± 0.8) compared to UC participants (*M *= 1.9 ± 0.8, *p *= 0.006).

Assessments of depression (PHQ-9), physical activity (Paffenbarger Physical Activity Scale) and general mental and physical functioning (SF-12) suggest that while mental health scores were generally within normal limits, on average, participants experienced some limitations in their physical functioning. The mean score on the Mental Component Summary (MCS) of the SF-12 was 48.0 ± 11.2. When comparing SF-12 MCS mean scores between groups, the UC group scored significantly lower (*M *= 47.1 ± 11.6) in overall mental health than the IC group (*M *= 48.7 ± 10.8, *p *= 0.03). Mean scores on PHQ-9 depression measure indicated that the UC group (*M *= 5.4 ± 4.7) scored in the mildly depressed range (PHQ-9 score ≥ 5) [21] and significantly higher than the IC group (*M *= 4.5 ± 4.6, *p *= 0.005). Approximately sixteen percent of the participants reached the cut-off for moderate/severe major depressive disorder (PHQ-9 score ≥ 10) [21]. The Physical Component Summary (PCS) score of the SF-12 was 42.3 ± 10.4, and the UC group scored significantly lower (*M *= 41.6 ± 10.9) on the scale than the IC group (*M *= 43.0 ± 9.8, *p *= 0.04). Paffenbarger Physical Activity Scale mean scores indicated that CAATCH participants were sedentary, burning on average 835.3 ± 1,644.2 Kcal per week in physical activity. While sleep quality scores (*M *= 7.0 ± 4.7) fell within the Epworth Sleepiness Scale (ESS) normal range (0 to 9) [20,29], one-fifth (20.9%) experienced problematic levels of daytime sleepiness, as indicated by scores of 10 or greater on the scale (see Table 5).

**Table 5:** Psychosocial and Behavioral Characteristics of Participants at Baseline

Characteristic	Total (n = 1,039)	Intervention Group (n = 529)	Usual Care Group (n = 510)	*p*
Medication Non-adherence (%; n = 961)	55.7	48.7	63.1	< 0.01**
Medication Adherence Self-Efficacy Scale (MASES; mean ± sd; n = 787)	2.2 ± 0.6	2.3 ± 0.6	2.2 ± 0.6	0.02*
Rapid Estimate of Adult Literacy in Medicine (REALM; mean ± sd; %; n = 889)	56.9 ± 13.2	57.3 ± 12.8	56.4 ± 13.6	0.28
3^rd ^Grade (0 to 18)	3.5	3.0	4.0	0.04*
4^th ^to 6^th ^Grade (19 to 44)	8.8	9.9	7.5	
7^th ^to 8^th ^Grade (45 to 60)	30.1	26.3	34.4	
9^th ^Grade and Greater (61 to 66)	57.6	60.8	54.1	
Rapid Eating Assessment for Patients (REAP; mean ± sd; n = 930)	1.8 ± 0.8	1.7 ± 0.8	1.9 ± 0.8	< 0.01**
Paffenbarger Physical Activity Scale (mean ± sd; %; n = 951)	835.3 Kcal ±	833 Kcal ±	837.8 Kcal ±	0.97
	1,644.2 Kcal	1,856.8 Kcal	1,388.9 Kcal	0.87
Sedentary (< 500 Kcal/week)	54.1	54.3	53.8	
Insufficiently Active (500 to 999 Kcal/week)	25.1	24.4	25.8	
Regularly Active (≥ 1,000 Kcal/week)	20.8	21.3	20.4	
Epworth Sleepiness Scale (mean ± sd; %; n = 949)	7.0 ± 4.7	6.9 ± 4.8	7.2 ± 4.6	0.46
Normal (≥ 10)	79.1	80.2	78.0	0.40
Excessive Daytime Sleepiness (> 10)	20.9	19.8	22.0	
Patient Health Questionnaire-9 (mean ± sd; %; n = 850)	4.9 ± 4.7	4.5 ± 4.6	5.4 ± 4.7	< 0.01**
Minimal (0 to 4)	56.2	59.4	52.8	0.28
Mild (5 to 9)	28.1	26.3	30.1	
Moderate (10 to 14)	10.9	10.4	11.5	
Moderately Severe (15 to 19)	4.1	3.2	5.1	
Severe (20 to 27)	0.6	0.7	0.5	
SF-12 (mean ± sd; n = 939)				
PCS-12	42.3 ± 10.4	43.0 ± 9.8	41.6 ± 10.9	0.04*
MCS-12	48.0 ± 11.2	48.7 ± 10.8	47.1 ± 11.6	0.03*

*Note*. Data gathered through interview. PCS-12 = Physical Component Summary; MCS-12 = Mental Component Summary. *p < .05; **p < 0.01

## Discussion

In this paper we describe the baseline characteristics of African American patients enrolled in the CAATCH trial, a cluster RCT multi-level, practice-based, BP control trial among a sample of CHCs with a rich diversity of demographic, clinical, psychosocial, and behavioral characteristics. Results from this trial will provide valuable information on interventions to promote BP control in a high-risk population of hypertensive African Americans with uncontrolled BP.

There were a number of patient-level barriers to hypertension control among the CAATCH sample at baseline, including low-income, significant co-morbidity, sedentary lifestyle, smoking, overweight, and obesity. This sample had a considerably lower mean income compared to patients in large-scale epidemiological studies, such as the multi-ethnic Dallas Heart Study [30,31] and the Jackson Heart Study [32,33]. When compared to other large-scale trials in hypertensive African Americans, CAATCH participants were more likely to be in particularly poor health. For example, CAATCH participants were heavier than those in the Dallas Heart Study [30], Jackson Heart Study [33], in Dietary Approaches to Stop Hypertension (DASH), Hypertension Improvement Project (HIP), and PREMIER trials [6,11,34]. Obesity rates reported for African Americans in the National Health and Nutrition Examination Survey (NHANES) were only 45% [35,36], compared to over two-thirds reported in CAATCH. Similar to published data on medication adherence [15,37-39], one-half of the CAATCH participants reported non-adherence, and they experienced diabetes and cardiovascular disease at rates higher than samples in large scale epidemiological trials such as the Jackson Heart Study [33]. In fact, CAATCH participants reported higher co-morbid medical conditions, which are recognized as significant barriers to BP control [40]. Physical activity levels in the CAATCH sample were very low, with most of the participants characterized as sedentary or insufficiently active. This a higher proportion than rates noted in the most recent NHANES data [41]. A noteworthy finding is that prevalence of smoking in the CAATCH trial was higher than those in the Jackson Heart Study [33], the Dallas Heart Study [30], and recent NHANES data on smoking among overweight and obese adults [41]. Further attention is warranted for several of the CAATCH sample's psychosocial and behavioral characteristics. CAATCH participants reported a moderate level of self-confidence in their ability to take antihypertensive medications (as measured by the MASES) [16], and a low level of healthy eating (as measured by the REAP) [18]. While the average general mental health score (Mental Component Summary of the SF-12) [22] of participants fell within normal range of population based norms, the UC participants scored in the mildly depressed range (as assessed by the PHQ-921). Furthermore, participants reported limitations in general physical functioning (Physical Component Summary of the SF-12) [22]. Though, on average, participants scored within the normal range on an index of sleep quality [20,29], problematic levels of daytime sleepiness (Epworth Sleep Scale score ≥ 10) were present among one-fifth. This rate is higher than the prevalence of daytime somnolence observed in recent NHANES data [42], yet lower than rates found in a recent analysis from the Sleep Heart Health Study [43]. The problematic daytime sleepiness observed in CAATCH participants may be due in part to the higher prevalence of obesity, which is a major risk factor for obstructive sleep apnea [44,45]. One-quarter of CAATCH participants had resistant hypertension (defined as any patient with uncontrolled BP who is taking at least three antihypertensive medications, including a diuretic). While thiazide type diuretics are the recommended initial pharmacological treatment [5,46], only one-half of the CAATCH sample had been prescribed one. However, this percentage is higher than the diuretic rate reported for a population of hypertensive patients from a large managed care organization, located in the southeastern United States, based on prescription fill data two years following publication of the JNC-7 guidelines [46].

One of the most striking aspects of the CAATCH trial is how its participants compare demographically and clinically to large-scale efficacy trials of lifestyle modification on BP levels [6,7,11,47], and four recently published effectiveness trials of BP control [34,48-51]. Participation of African Americans in previous efficacy trials ranged from 25 to 50% of the sample [6,7,11,34,49,51], while the CAATCH trial sample consists entirely of African American participants. Of particular note, the CAATCH trial represents a substantive improvement in African American male participation over previous trials in that almost one-third of participants were African American men. By contrast, one-tenth of participants in PREMIER were African American men. To our knowledge, the CAATCH trial includes the largest proportion of African American men in community practice-based trials. Because trust is a major hindrance to recruitment of minorities into clinical research [52], we adopted a two-pronged strategy to increase the participation of African American men, in addition to the traditional compensation provided to patients. First, we solicited the support of the CHC patient care coordinators and clerical staff in referring patients into the study. CHC staff typically interacts with patients over a longer period of time and have built significant rapport and trust with the patients, whereas research staff have a shorter duration and more circumscribed relationship with prospective study participations. Second, we asked the clinicians to refer their patients into the trial. This strategy also addresses the issue of trust, building on the existing relationships between patients and providers. In addition, we oversampled African American men, because women comprise 70% of the adult users of CHCs. Finally, we held series of educational sessions at the study sites to inform patients about the study and to identify and screen potentially eligible patients; refreshments were offered at these sessions.

To our knowledge, CAATCH is the largest practice-based trial of patient and physician targeted interventions for BP control in hypertensive African Americans. We are only aware of three other trials that have targeted both patients and physicians in practice-based settings [34,49,50]. The first is the HIP trial [34], which is a nested, 2 × 2 RCT comparing physician intervention, patient intervention, and both combined versus control condition, among 574 hypertensive patients in eight primary care practices (32 physicians) in central North Carolina. The second is the Veterans Study to Improve the Control of Hypertension (V-STITCH) trial [49], which is a 2-level (primary care provider and patient) cluster RCT among 588 hypertensive patients in a Veterans Affairs Medical Center primary care clinic (17 physicians) in North Carolina. The third is a study by Roumie *et al*., which is a cluster RCT [50] that evaluated the impact of three quality improvement interventions (provider education, provider education and alert, patient education) of increasing intensity among 1,341 hypertensive patients who received care in two hospital-based and eight community-based outpatient clinics (182 providers) in the Veterans Affairs Tennessee Valley Healthcare System. One qualitative difference between CAATCH and these trials is that CAATCH was focused exclusively on African Americans. Second, the patients in CAATCH all had uncontrolled hypertension and greater co-morbidity than those in the other practice-based trials. Specifically, CAATCH participants had higher baseline BP, were more sedentary, had greater levels of co-morbidity, and higher current smoking rates than participants in the V-STITCH and HIP trials [34,49]. Furthermore, CAATCH participants had comparable rates of smoking and higher rates of diabetes, and lower rates of medication adherence than participants in Roumie *et al*. VA-based trial of interventions targeting providers and patients [50]. Perhaps of even more interest, participants in the CAATCH trial had lower income than those in the HIP, V-STITCH, DASH, and PREMIER trials, had less education than participants in DASH, and PREMIER, and as a group, were more likely to be underinsured. Thus, the patient population in CAATCH is more representative of low-income African Americans with poorly controlled hypertension and numerous adverse lifestyle behaviors and clinical characteristics that constitute barriers to adequate BP control. As such, the findings from CAATCH will provide needed information on effectiveness of lifestyle and self-management approaches in care of this high-risk population in a more generalizable context. Furthermore, the participating CHCs are distributed widely across upstate New York, four boroughs of New York City, Northern New Jersey, and serve low-income, African American patient populations. Utilization of EHRs among the participating sites at the time of recruitment and intervention was relatively low, and approximately one-half of the CHCs participating in the trial were involved in the HRSA Health Disparities Collaborative, an initiative that focuses on improving access to quality healthcare and improved health outcomes among medically underserved in order to reduce or eliminate health disparities [53].

Several strengths of the CAATCH trial deserve consideration. The first is that CAATCH is an evidence-based multi-level intervention in a very large exclusively African American practice-based sample, in contrast to previous efficacy and recent effectiveness trials in which the rate of African American participation has been more modest [6,7,11,34,48,54]. Second, CAATCH inclusion criteria allowed for the participation of patients with uncontrolled BP and those taking multiple medications, while the exclusion criteria of previous efficacy studies [6,7,11] were more conservative by comparison. Third, CAATCH joins a small number of effectiveness trials (implemented in the VA [49] or community based primary care clinics [34]) in efforts to deliver efficacious treatments in real-world primary care settings. CAATCH is one of few studies to address barriers to BP control in this patient population by evaluating the effectiveness of a practice-based intervention targeted at patients (home BP monitoring, lifestyle intervention plus patient education) and physicians (chart audit and feedback plus physician education). Thus, findings from this trial will extend our understanding of applicability and impact of a multi-level intervention in a population whose hypertension may be particularly difficult to control.

We should note the following limitations of the CAATCH trial. First, similar to other large scale RCTs in African Americans, majority of CAATCH participants were women, making the results less generalizable to African American men. Second, there were imbalances in several important baseline patient characteristics between the IC and UC study sites, which raises potential threats to internal validity of the study findings. The cluster randomized design of the study sought to minimize baseline differences between both groups via matching of CHCs based on practice size. The reason for the observed baseline differences is not clear. One potential explanation for this imbalance may be the small number of sites that were randomized rather than the number of participants. Although the intraclass correlation coefficient (ICC) between the sites on these variables was quite small [24,55], the additional matching of sites by size may have required larger number of sites than the proposed 30 sites. Alternative approaches to mitigate this problem maybe the use of a factorial design with randomization at the level of the providers on characteristics, such as number of years practicing in a community-based setting, size of caseload, and number of hypertensive patients in current caseload. This is the approach adopted by Corsino *et al*. [56], which often requires matching on lesser number of variables than sites and manageable number of providers and patients needed for the study. Other approaches of randomizing at the patient level with matching may also mitigate this problem, but such an approach is limited by the potential for contamination that is often a problematic in practice-based trials using such design, and the loss of the ability to examine the contribution of matching variables to changes in study outcomes.

In summary, the demographic and clinical composition of the CAATCH trial participants represents an important contribution to the literature on interventions targeted at poor BP control in a high-risk population. Namely, the trial includes implementation of a multi-level, evidence-based, intervention in a real-world setting among participants who experience multiple demographic, clinical, psychosocial, and behavioral barriers to hypertension control. Findings from this trial have important implications for dissemination and implementation. First, successful conduct of this study will be an indication that complex multi-level intervention can be integrated into care of high-risk hypertensive African Americans who receive care in CHCs. Second, imbalance noted in the baseline data indicates that investigators considering cluster RCT design in this patient population should strongly consider enrolling a larger number of sites and possibly an alternative design with randomization at the provider level. Third, recruitment of this high-risk patient population was achieved with significant cooperation from the clinical and administrative staff at the participating CHCs. A major lesson learned in this regard is the underlying assumption that all CHCs do not operate in the same manner. While their payment structure may be similar, the staffing in these sites is quite varied in terms of the use of allied health providers and availability of health educators who can provide lifestyle counseling and patient education. Furthermore, the level of enthusiasm for pragmatic trials is quite different for each CHC, with some having more research experience and others lacking experience. This factor affected recruitment rates, follow-up rates, and delivery of the intervention. Specifically, sites that were more research-friendly had the highest completion rates with easier delivery of the intervention. Future practice-based research should better characterize the research sites along these dimensions in order to facilitate implementation of such complex studies and to assess the interaction between aspects of study implementation and CHC trial characteristics. Fourth, implementation of the patient level component of the intervention, including lifestyle counseling, could be strengthened by a flexible delivery format. For example, we found that a combination of in-person intervention sessions coupled with telephone check-in or booster sessions to support patient up-take of new skills allowed us to improve treatment fidelity levels.

## Conclusions

The CAATCH trial is the first of its kind to focus on the effectiveness of a multi-level intervention for BP control in a large, socio-demographically and clinically diverse sample of hypertensive African American patients receiving primary care in safety-net settings. Given characteristics of the CHCs and participants, CAATCH has the potential to extend our understanding of barriers and facilitators of BP control in a group that experiences significant hypertension-related morbidity and mortality.

## Competing interests

The authors declare that they have no competing interests.

## Authors' contributions

SF participated in the design, coordination and analysis of the study. AC, MDG, and CK participated in the coordination of the study. GO and JNT conceived of the study and participated in the design, coordination, and analysis of the study. All authors contributed to the preparation of the manuscript, and read and approved the final manuscript.

## References

[B1] Fields LE Burt VL Cutler JA Hughes J Roccella EJ Sorlie P The burden of adult hypertension in the United States 1999 to 2000: a rising tide Hypertension 2004 44 398 404 10.1161/01.HYP.0000142248.54761.56 15326093

[B2] Cutler JA Sorlie PD Wolz M Wolz M Thom T Fields LE Roccella EJ Trends in hypertension prevalence, awareness, treatment, and control rates in United States adults between 1988-1994 and 1999-2004 Hypertension 2008 52 5 818 827 10.1161/HYPERTENSIONAHA.108.113357 18852389

[B3] Giles T Aranda JM Jr Suh DC Choi IS Preblick R Rocha R Frech-Tamas F Ethnic/racial variations in blood pressure awareness, treatment, and control Journal of Clinical Hypertension 2007 9 5 345 354 10.1111/j.1524-6175.2007.06432.x 17485970 PMC8109924

[B4] Gu Q Burt VL Paulose-Ram R Yoon S Gillum RF High blood pressure and cardiovascular disease mortality risk among U.S. adults: the third National Health and Nutrition Examination Survey mortality follow-up study.[erratum appears in Ann Epidemiol. 2008 Jun;18(6):515] Annals of Epidemiology 2008 18 4 302 309 10.1016/j.annepidem.2007.11.013 18261929

[B5] Chobanian AV Bakris GL Black HR Cushman WC Green LA Izzo JL Jr Jones DW Materson BJ Oparil S Wright JT Jr Roccella EJ Seventh report of the Joint National Committee on Prevention, Detection, Evaluation, and Treatment of High Blood Pressure Hypertension 2003 42 6 1206 1252 10.1161/01.HYP.0000107251.49515.c2 14656957

[B6] Appel LJ Champagne CM Harsha DW Cooper LS Obarzanek E Elmer PJ Stevens VJ Vollmer WM Lin PH Svetkey LP Stedman SW Young DR Effects of comprehensive lifestyle modification on blood pressure control: main results of the PREMIER clinical trial JAMA 2003 289 16 2083 2093 10.1001/jama.289.16.2083 12709466

[B7] Appel LJ Espeland MA Easter L Wilson AC Folmar S Lacy CR Effects of reduced sodium intake on hypertension control in older individuals: results from the Trial of Nonpharmacologic Interventions in the Elderly (TONE) Arch Intern Med 2001 161 5 685 693 10.1001/archinte.161.5.685 11231700

[B8] Boulware LE Daumit GL Frick KD Minkovitz CS Lawrence RS Powe NR An evidence-based review of patient-centered behavioral interventions for hypertension Am J Prev Med 2001 21 3 221 232 10.1016/S0749-3797(01)00356-7 11567845

[B9] Fahey T Schroeder K Ebrahim S Interventions used to improve control of blood pressure in patients with hypertension Cochrane Database Syst Rev 2006 2 CD005182 10.1002/14651858.CD005182.pub2 16625627

[B10] Sacks FM Svetkey LP Vollmer WM Appel LJ Bray GA Harsha D Obarzanek E Conlin PR Miller ER Simons-Morton DG Karanja N Lin PH Effects on blood pressure of reduced dietary sodium and the Dietary Approaches to Stop Hypertension (DASH) diet. DASH-Sodium Collaborative Research Group N Engl J Med 2001 344 1 3 10 10.1056/NEJM200101043440101 11136953

[B11] Appel LJ Moore TJ Obarzanek E Vollmer WM Svetkey LP Sacks FM Bray GA Vogt TM Cutler JA Windhauser MM Lin PH Karanja N A clinical trial of the effects of dietary patterns on blood pressure. DASH Collaborative Research Group N Engl J Med 1997 336 16 1117 1124 10.1056/NEJM199704173361601 9099655

[B12] Ogedegbe G Tobin JN Fernandez S Gerin W Diaz-Gloster M Cassells A Khalida C Pickering T Schoenthaler A Ravenell J Counseling African Americans to Control Hypertension (CAATCH) trial: A multi-level intervention to improve blood pressure control in hypertensive Blacks Circulation: Cardiovascular Quality and Outcomes 2009 2 249 256 10.1161/CIRCOUTCOMES.109.849976 PMC2800792 20031845

[B13] Altman DG Schulz KF Moher D Egger M Davidoff F Elbourne D Gøtzsche PC Lang T The revised CONSORT statement for reporting randomized trials: explanation and elaboration Ann Intern Med 2001 134 8 663 694 11304107 10.7326/0003-4819-134-8-200104170-00012

[B14] Perloff D Grim C Flack J Frohlich ED Hill M McDonald M Morgenstern BZ Human blood pressure determination by sphygmomanometry Circulation 1993 88 5 Pt 1 2460 2470 8222141 10.1161/01.cir.88.5.2460

[B15] Morisky DE Green LW Levine DM Concurrent and predictive validity of a self-reported measure of medication adherence Med Care 1986 24 1 67 74 10.1097/00005650-198601000-00007 3945130

[B16] Ogedegbe G Mancuso CA Allegrante JP Charlson ME Development and evaluation of a medication adherence self-efficacy scale in hypertensive African-American patients J Clin Epidemiol 2003 56 6 520 529 10.1016/S0895-4356(03)00053-2 12873646

[B17] Davis TC Crouch MA Long SW Jackson RH Bates P George RB Bairnsfather LE Rapid assessment of literacy levels of adult primary care patients Fam Med 1991 23 6 433 435 1936717

[B18] Gans KM Ross E Barner CW Wylie-Rosett J McMurray J Eaton C REAP and WAVE: New Tools to Rapidly Assess/Discuss Nutrition with Patients Journal of Nutrition 2003 133 556S 562S 12566502 10.1093/jn/133.2.556S

[B19] Paffenbarger RS Blair SN Lee IM Hyde RT Measurement of physical activity to assess health effects in free-living populations Med Sci Sports Exerc 1993 25 1 60 70 10.1249/00005768-199301000-00010 8423758

[B20] Johns MW A new method for measuring daytime sleepiness: the Epworth sleepiness scale Sleep 1991 14 6 540 545 1798888 10.1093/sleep/14.6.540

[B21] Kroenke K Spitzer RL Williams JB The PHQ-9: validity of a brief depression severity measure J Gen Intern Med 2001 16 9 606 613 10.1046/j.1525-1497.2001.016009606.x 11556941 PMC1495268

[B22] Ware JE Kosinski M Keller SD A 12-Item Short-Form Health Survey: Construction of scales and preliminary tests of reliability and validity Medical Care 1996 34 3 220 233 10.1097/00005650-199603000-00003 8628042

[B23] Wagner EH Austin BT Davis C Hindmarsh M Schaefer J Bonomi A Improving chronic illness care: translating evidence into action Health Aff (Millwood) 2001 20 6 64 78 10.1377/hlthaff.20.6.64 11816692

[B24] Gerin W Tobin JN Schwartz JE Chaplin W Rieckmann N Davidson KW Goyal TM Jhalani J Cassells A Feliz K Khalida C Diaz-Gloster M Ogedegbe G The medication Adherence and Blood Pressure Control (ABC) trial: a multi-site randomized controlled trial in a hypertensive, multi-cultural, economically disadvantaged population Contemporary Clinical Trials 2007 28 4 459 471 10.1016/j.cct.2007.01.003 17287150

[B25] SAS System for Mixed Models [computer program] Version Cary, NC: SAS Institute Inc

[B26] SAS Technical Report P-229, SAS/STAT Software: Changes and Enhancements [computer program] Version 1992 Cary, NC: SAS Institute Inc

[B27] Hedeker D Gibbons RD A random-effects ordinal regression model for multilevel analysis Biometrics 1994 50 4 933 944 10.2307/2533433 7787006

[B28] Hedeker D Gibbons RD MIXOR: a computer program for mixed-effects ordinal regression analysis Comput Methods Programs Biomed 1996 49 2 157 176 10.1016/0169-2607(96)01720-8 8735023

[B29] Johns M Hocking B Daytime sleepiness and sleep habits of Australian workers Sleep 1997 20 10 844 849 9415943 10.1093/sleep/20.10.844

[B30] Victor RG Haley RW Willett DL Peshock RM Vaeth PC Leonard D Basit M Cooper RS Iannacchione VG Visscher WA Staab JM Hobbs HH The Dallas Heart Study: a population-based probability sample for the multidisciplinary study of ethnic differences in cardiovascular health American Journal of Cardiology 2004 93 12 1473 1480 10.1016/j.amjcard.2004.02.058 15194016

[B31] Albert MA Ravenell J Glynn RJ Khera A Halevy N de Lemos JA Cardiovascular risk indicators and perceived race/ethnic discrimination in the Dallas Heart Study American Heart Journal 2008 156 6 1103 1109 10.1016/j.ahj.2008.07.027 19033005

[B32] Taylor HA Jr Wilson JG Jones DW Sarpong DF Srinivasan A Garrison RJ Nelson C Wyatt SB Toward resolution of cardiovascular health disparities in African Americans: design and methods of the Jackson Heart Study Ethnicity & Disease 2005 15 4 Suppl 6 S6 4-17 16320381

[B33] Wyatt SB Akylbekova EL Wofford MR Coady SA Walker ER Andrew ME Keahey WJ Taylor HA Jones DW Prevalence, awareness, treatment, and control of hypertension in the Jackson Heart Study Hypertension 2008 51 3 650 656 10.1161/HYPERTENSIONAHA.107.100081 18268140

[B34] Svetkey LP Pollak KI Yancy WS Jr Dolor RJ Batch BC Samsa G Matchar DB Lin PH Hypertension improvement project: randomized trial of quality improvement for physicians and lifestyle modification for patients Hypertension 2009 54 6 1226 1233 10.1161/HYPERTENSIONAHA.109.134874 19920081 PMC2784648

[B35] Flegal KM Carroll MD Ogden CL Curtin LR Prevalence and trends in obesity among US adults, 1999-2008 JAMA 303 3 235 241 Jan 20; 10.1001/jama.2009.2014 20071471

[B36] Ogden CL Carroll MD Curtin LR McDowell MA Tabak CJ Flegal KM Prevalence of overweight and obesity in the United States, 1999-2004 JAMA 2006 295 13 1549 1555 10.1001/jama.295.13.1549 16595758

[B37] Haynes RB Ackloo E Sahota N McDonald HP Yao X Interventions for enhancing medication adherence Cochrane Database Syst Rev 2008 2 CD000011 10.1002/14651858.CD000011.pub3 18425859

[B38] Ogedegbe G Chaplin W Schoenthaler A Statman D Berger D Richardson T Phillips E Spencer J Allegrante JP A practice-based trial of motivational interviewing and adherence in hypertensive African Americans Am J Hypertens 2008 21 10 1137 1143 10.1038/ajh.2008.240 18654123 PMC3747638

[B39] Osterberg L Blaschke T Osterberg L Blaschke T Adherence to medication New England Journal of Medicine 2005 353 5 487 497 10.1056/NEJMra050100 16079372

[B40] Wong ND Lopez VA L'Italien G Chen R Kline SE Franklin SS Inadequate control of hypertension in US adults with cardiovascular disease comorbidities in 2003-2004 Archives of Internal Medicine 2007 167 22 2431 2436 10.1001/archinte.167.22.2431 18071164

[B41] McDowell MA Hughes JP Borrud LG McDowell MA Hughes JP Borrud LG Health characteristics of U.S. adults by body mass index category: results from NHANES 1999-2002 Public Health Reports 2006 121 1 67 73 Reprint in MMWR Morb Mortal Wkly Rep. 2006 Jun 16;55(23):656; PMID: 16789325 16416700 10.1177/003335490612100113 PMC1497803

[B42] Qureshi AI Giles WH Croft JB Bliwise DL Habitual sleep patterns and risk for stroke and coronary heart disease: a 10-year follow-up from NHANES I Neurology 1997 48 4 904 911 9109875 10.1212/wnl.48.4.904

[B43] Kapur VK Resnick HE Gottlieb DJ Sleep disordered breathing and hypertension: does self-reported sleepiness modify the association? Sleep 2008 31 8 1127 1132 18714785 PMC2542959

[B44] Young T Peppard PE Gottlieb DJ Young T Peppard PE Gottlieb DJ Epidemiology of obstructive sleep apnea: a population health perspective American Journal of Respiratory & Critical Care Medicine 2002 165 9 1217 1239 10.1164/rccm.2109080 11991871

[B45] Young T Skatrud J Peppard PE Young T Skatrud J Peppard PE Risk factors for obstructive sleep apnea in adults JAMA 2004 291 16 2013 2016 10.1001/jama.291.16.2013 15113821

[B46] Muntner P Krousel-Wood M Hyre AD Stanley E Cushman WC Cutler JA Piller LB Goforth GA Whelton PK Antihypertensive prescriptions for newly treated patients before and after the main antihypertensive and lipid-lowering treatment to prevent heart attack trial results and seventh report of the joint national committee on prevention, detection, evaluation, and treatment of high blood pressure guidelines Hypertension 2009 53 4 617 623 10.1161/HYPERTENSIONAHA.108.120154 19221214

[B47] Svetkey LP Harsha DW Vollmer WM Stevens VJ Obarzanek E Elmer PJ Lin PH Champagne C Simons-Morton DG Aickin M Proschan MA Appel LJ Premier: a clinical trial of comprehensive lifestyle modification for blood pressure control: rationale, design and baseline characteristics Ann Epidemiol 2003 13 6 462 471 10.1016/S1047-2797(03)00006-1 12875806

[B48] Dolor RJ Yancy WS Jr Owen WF Matchar DB Samsa GP Pollak KI Lin PH Ard JD Prempeh M McGuire HL Batch BC Fan W Svetkey LP Hypertension Improvement Project (HIP): study protocol and implementation challenges Trials 2009 10 13 10.1186/1745-6215-10-13 19245692 PMC2654882

[B49] Bosworth HB Olsen MK Dudley T Orr M Goldstein MK Datta SK McCant F Gentry P Simel DL Oddone EZ Patient education and provider decision support to control blood pressure in primary care: a cluster randomized trial American Heart Journal 2009 157 3 450 456 10.1016/j.ahj.2008.11.003 19249414

[B50] Roumie CL Elasy TA Greevy R Griffin MR Liu X Stone WJ Wallston KA Dittus RS Alvarez V Cobb J Speroff T Improving blood pressure control through provider education, provider alerts, and patient education: a cluster randomized trial Annals of Internal Medicine 2006 145 3 165 175 16880458 10.7326/0003-4819-145-3-200608010-00004

[B51] Bosworth HB Olsen MK Grubber JM Neary AM Orr MM Powers BJ Adams MB Svetkey LP Reed SD Li Y Dolor RJ Oddone EZ Two Self-management Interventions to Improve Hypertension Control Annals of Internal Medicine 2009 10.1059/0003-4819-151-10-200911170-00148 PMC2892337 19920269

[B52] Ford JG Howerton MW Lai GY Gary TL Bolen S Gibbons MC Tilburt J Baffi C Tanpitukpongse TP Wilson RF Powe NR Bass EB Barriers to recruiting underrepresented populations to cancer clinical trials: A systematic review Cancer 2008 112 2 228 242 10.1002/cncr.23157 18008363

[B53] Hicks LS O'Malley AJ Lieu TA Keegan T McNeil BJ Guadagnoli E Landon BE Impact of health disparities collaboratives on racial/ethnic and insurance disparities in US community health centers Archives of Internal Medicine 2010 170 3 279 286 10.1001/archinternmed.2010.493 20142575

[B54] Bosworth HB Olsen MK Dudley T Orr M Goldstein MK Datta SK McCant F Gentry P Simel DL Oddone EZ Patient education and provider decision support to control blood pressure in primary care: A cluster randomized trial American Heart Journal 2009 157 3 450 456 10.1016/j.ahj.2008.11.003 19249414

[B55] Schoenthaler A Chaplin WF Allegrante JP Fernandez S Diaz-Gloster M Tobin JN Ogedegbe G Provider communication effects medication adherence in hypertensive African Americans Patient education and counseling 2009 75 2 185 191 10.1016/j.pec.2008.09.018 19013740 PMC2698021

[B56] Corsino L Yancy WS Samsa GP Dolor RJ Pollak KI Lin PH Svetkey LP Physician Characteristics as Predictors of Blood Pressure Control in Patients Enrolled in the Hypertension Improvement Project (HIP) J Clin Hypertens (Greenwich) 2011 13 2 106 111 10.1111/j.1751-7176.2010.00385.x PMC3333480 21272198

